# Localization of epitopes recognized by monoclonal antibodies that neutralized the H3N2 influenza viruses in man

**DOI:** 10.1099/vir.0.026419-0

**Published:** 2011-02

**Authors:** Jun Okada, Nobuko Ohshima, Ritsuko Kubota-Koketsu, Yoshitaka Iba, Sayuri Ota, Wakana Takase, Tetsushi Yoshikawa, Toyokazu Ishikawa, Yoshizo Asano, Yoshinobu Okuno, Yoshikazu Kurosawa

**Affiliations:** 1 Division of Antibody Project, Institute for Comprehensive Medical Science, Fujita Health University, Toyoake, Aichi 470-1192, Japan; 2 Institute for Antibody Ltd, Toyoake, Aichi 470-1192, Japan; 3 21st Century COE Research Center, Fujita Health University, Toyoake, Aichi 470-1192, Japan; 4 Department of Virology, Research Institute for Microbial Diseases, Osaka University, Suita, Osaka 565-0871, Japan; 5 Department of Pediatrics, School of Medicine, Fujita Health University, Toyoake, Aichi 470-1192, Japan; 6 The Research Foundation for Microbial Diseases, Osaka University, Kannonji, Kagawa 768-0061, Japan

## Abstract

Through extensive isolation of neutralizing mAbs against H3N2 influenza viruses representing the *in vivo* repertoire in a human donor, we examined the relationships between antigenic drift of influenza virus and protective antibodies generated in an infected individual. The majority of mAbs isolated from a donor born in 1960 were divided into three major groups with distinct strain specificity: 1968–1973, 1977–1993 and 1997–2003. In the present study, we developed a new method that allowed us to comprehensively determine the location of epitopes recognized by many mAbs. Original haemagglutinins (HAs) of several strains and chimaeric variants, in which one of the seven sites (A, B1, B2, C1, C2, D or E) was replaced by some other strain-derived sequence, were artificially expressed on the cell surface. The binding activity of mAbs to the HAs was examined by flow cytometry. By using this method, we determined the location of epitopes recognized by 98 different mAbs. Clones that neutralize the 1968–1973 strains bind to site B2/D, A or A/B1. While sites C, E and B were recognized by clones that neutralized the 1977–1993 strains, the majority of these clones bind to site C. Clones that neutralize the 1997–2003 strains bind to site B, A/B1, A/B2 or E/C2.

## INTRODUCTION

Antibodies (Abs) play important roles in protection against and recovery from influenza virus infection, and haemagglutinin (HA) is the main target for virus-neutralizing Abs (Gerhard *et al.*, 1997). Viruses with mutations in critical residues of HA could preferentially survive under the pressure of neutralizing Abs and cause future influenza epidemics (Hay *et al.*, 2001). Mutations mainly accumulate in the five sites (A, B, C, D and E) on HA1 that should include neutralizing epitopes (Underwood, 1982; Wiley *et al.*, 1981). To the best of our knowledge, however, very little is known about the nature, range and specificity of protective mAbs that are generated in infected individuals. Since the outbreak of ‘Hong Kong’ influenza in 1968, the H3N2 influenza viruses remain the most common cause of flu. Therefore, in the present study, we examined the neutralizing Abs against the H3 strains isolated between 1968 and 2004.

We attempted to reveal the relationships between the location of epitopes recognized by neutralizing mAbs against influenza viruses in a human donor and that of mutations introduced in the epidemic strains. Previously (Okada *et al.*, 2010), we collected a large number of B lymphocytes by apheresis from a donor born in 1960. Since the number of collected B cells was estimated to be around 10^9^, they probably represented 0.1–1 % of his total B-cell population. By using phage-display technology (Winter *et al.*, 1994), we constructed a large Ab library composed of 3×10^10^ clones. The library was screened against inactivated virus particles of 12 different vaccine strains isolated between 1968 and 2004. The clones that bound to the antigens (Ags) were isolated, and mAbs that specifically bound to H3-strain viruses were selected. Finally, their binding activity to the 12 strains and neutralizing activity were studied. The 1143 clones were analysed. The sequence analysis revealed that they were composed of 153 unique mAbs with different V_H_ sequences. Of the 153 clones, 113 showed both binding activity and virus-neutralizing activity, while the remaining 40 clones showed binding activity but did not show neutralizing activity. The majority of clones with neutralizing activity were anti-HA Abs that could be divided into three major groups showing distinct strain specificity: 1968–1973, 1977–1993 and 1997–2003. In the present study, we analysed the location of the epitopes recognized by these clones.

## RESULTS

### A new method for identifying the location of epitopes recognized by mAbs with neutralizing activity

**Fig. 1.**
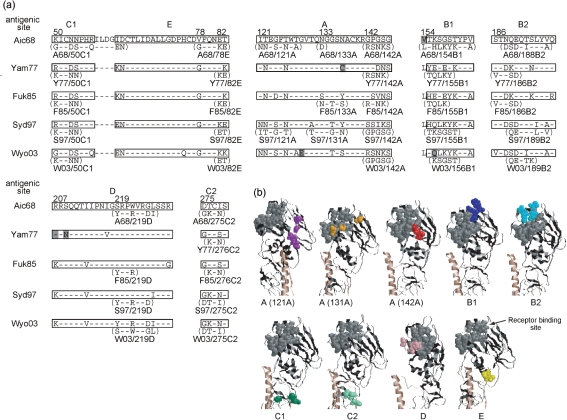
Amino acid sequences of the seven antigenic sites in template and chimaeric HAs constructed in this study. (a) HAs of five vaccine strains, Aichi/68 (Aic68), Yamanashi/77 (Yam77), Fukuoka/85 (Fuk85), Sydney/97 (Syd97), Wyoming/2003 (Wyo03), were used as starting materials. The deduced amino acid sequences showed discrepancies with the reported data (Okada *et al.*, 2010) at six residues, which are highlighted in grey. Since we used these plasmids for the expression of HA the actual sequence determined is indicated in this figure. The name of the chimaeric HA and the amino acids that replaced the original ones are indicated under the template HA. Bars indicate the same amino acids as those of Aichi/68. (b) Location of the amino acids replaced in the chimaeric HA is indicated in the 3D structure of HA by using the examples of the Sydney/97 strain. The amino acids that formed a receptor-binding site were marked in grey. The amino acids replaced in the chimaera were marked as follows: site A, purple (121A), orange (131A) or red (142A); B1, blue; B2, light blue; C1, green; C2, light green; D, pink; E, yellow.

**Fig. 2.**
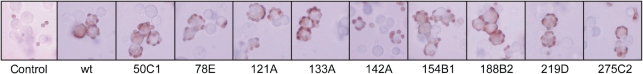
Attachment of reticulocytes to the cells expressing wild-type (wt) or chimaeric HAs of Aichi/68 influenza strain. 293T cells were transfected with plasmid containing wt or chimaeric HA genes of the Aichi/68 stain and incubated with guinea-pig red blood cells. As a control, plasmid without the HA gene was used.

Although epitopes could be identified by analyses of escape mutants that can grow in the presence of neutralizing mAb (Gerhard *et al.*, 1981), this method is time-consuming and labour-intensive, especially since we wanted to identify the epitopes recognized by many mAbs. Therefore, we developed a simpler method for epitope mapping that is based on the analysis of chimaeras. After HA was artificially expressed on the cell surface, the binding activity of respective mAbs to HA was examined by flow cytometry (FCM). Since the range of specificity of the majority of clones was 1968–1973, 1977–1993 or 1997–2003 (Okada *et al.*, 2010), HAs of the following five strains were selected: A/Aichi/2/68, A/Yamanashi/2/77, A/Fukuoka/C29/85, A/Sydney/5/97 and A/Wyoming/3/2003. To identify the epitope, several chimaeric HA variants for each strain were constructed. We assumed that the epitopes would be localized in one of the seven sites (A, B1, B2, C1, C2, D or E), and we constructed chimaeric variants in which one of the seven sites was replaced by some other strain-derived sequence. Fig. 1(a) summarizes the amino acid sequences of the original five HAs and their chimaeric variants, and Fig. 1(b) shows the three-dimensional (3D) location of amino acid residues changed in the chimaeric HA of the Sydney/97 strain. In the case of the Aichi/68 strain, the original HA and nine kinds of chimaeric HA were constructed. To determine if the 3D structures of HAs artificially expressed on the cell surface were properly formed, the haemadsorption activity of the transfected cells was examined. As shown in Fig. 2, several reticulocytes were attached to cells expressing the original HA and nine chimaeric HAs of the Aichi/68 strain. Essentially the same results were obtained for the other four strain-derived HA-transfected cells, with the exception of S97/50C1. These results indicate that the receptor-binding sites of HAs expressed on the cell surface were properly formed.

**Fig. 3.**
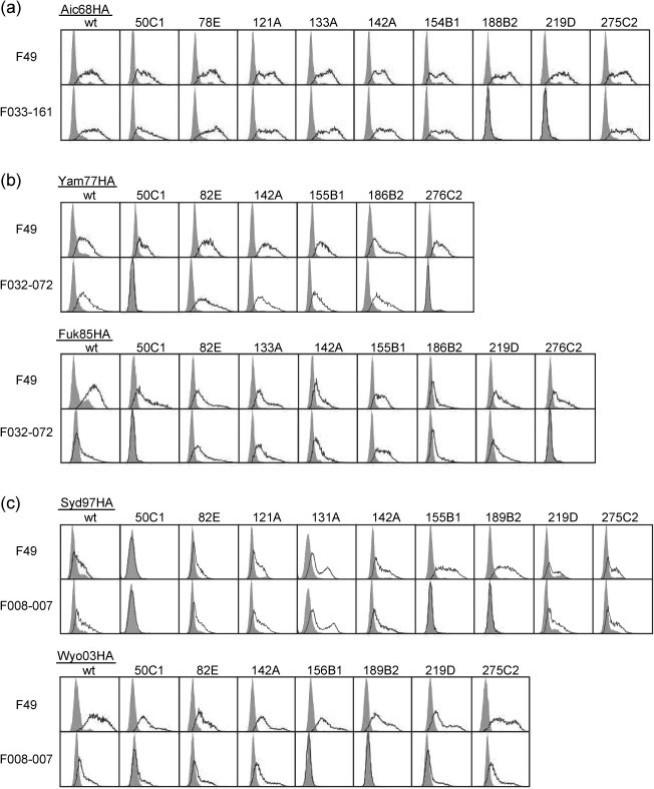
FCM analyses of wt and chimaeric HAs of five influenza strains. F49 is a mouse mAb that recognizes an epitope commonly present on HA of H3 subtype influenza viruses (Hay *et al.*, 2001). The Fab-PP form of Ab was used for F033-161 (a), F032-072 (b) and F008-007 (c).

To further examine whether these constructs could function as Ags, we performed pilot experiments with three representative clones: F033-161, F032-072 and F008-007. Fig. 3 shows the FCM profiles. Clone F033-161, which binds and neutralizes the 1968–1973 strains, was tested for its ability to bind the HAs of the Aichi/68 strains (Fig. 3a). Clone F49, which binds to the epitope commonly present on HAs of all the H3 strains, was used as a positive control (Ueda *et al.*, 1998). Clone F49 bound to the original HA-transfected cells and the nine chimaeric HA-transfected cells, suggesting that all of the HAs were expressed on the cell surface. F033-161 did not bind to either A68/188B2- or A68/219D-encoding DNA-transfected cells, but it did bind to all of the other HA-encoding DNA-transfected cells. These results suggest that F033-161 could not bind to HAs encoded by A68/188B2 and A68/219D, although they were present on the cell surface. We interpreted these results as evidence showing that the epitope recognized by clone F033-161 is located in site B2/D. Clone F032-072 was also tested (Fig. 3b). Since it binds and neutralizes the 1977–1993 strains, HAs of Yamanashi/77 and Fukuoka/85 were used as binding targets. The results indicate that F032-072 does not bind to Y77/50C1, Y77/276C2, F85/50C1 or F85/276C2; however, it does bind to all of the other HAs. We concluded that clone F032-072 bound to site C1/C2. Finally, clone F008-007 was examined (Fig. 3c). Since it binds and neutralizes the 1997–2003 strains, HAs of Sydney/97 and Wyoming/2003 were used as binding targets. F49 did not react with S97/50C1; therefore, we determined that this chimaeric HA molecule was not properly expressed on the cell surface. F008-007 did not bind to S97/155B1, S97/189B2 or S97/50C1. Since F008-007 also did not bind to W03/156B1 or W03/189B2 but did bind to all of the other HAs of the Sydney/97 and Wyoming/2003 strains, we concluded that site B1/B2 may contain the epitope recognized by this clone.

Based on the FCM results from the chimaeric HAs together with the examination of their haemadsorption activity, we concluded that the HA constructs of the five H3 strains, with the exception of S97/50C1, could function as Ags expressed on the cell surface and that the epitopes of these Ags could be recognized by respective mAbs. Thus, we termed this method EMAC (epitope mapping through analysis of chimaeras).

### Location of epitopes recognized by 98 mAbs that neutralize H3N2 strains

We used the EMAC method to examine epitopes recognized by mAbs with neutralizing activity. Ninety-eight clones that had been classified into 27 groups (Okada *et al.*, 2010) were divided into three sets. The first set consisted of 11 clones (classified into groups 1–3) that bound to the 1968–1973 strains. The second set consisted of four clones (classified into groups 4 and 5) that bound to the 1977–1999 strains and 54 clones (classified into groups 6–19) that bound to the 1977–1993 strains. The third set consisted of 14 clones (classified into groups 20 and 21) that bound to the 1993–2003 strains and 15 clones (classified into groups 23–27 and 29) that bound to the 1997–2003 strains.

**Fig. 4.**
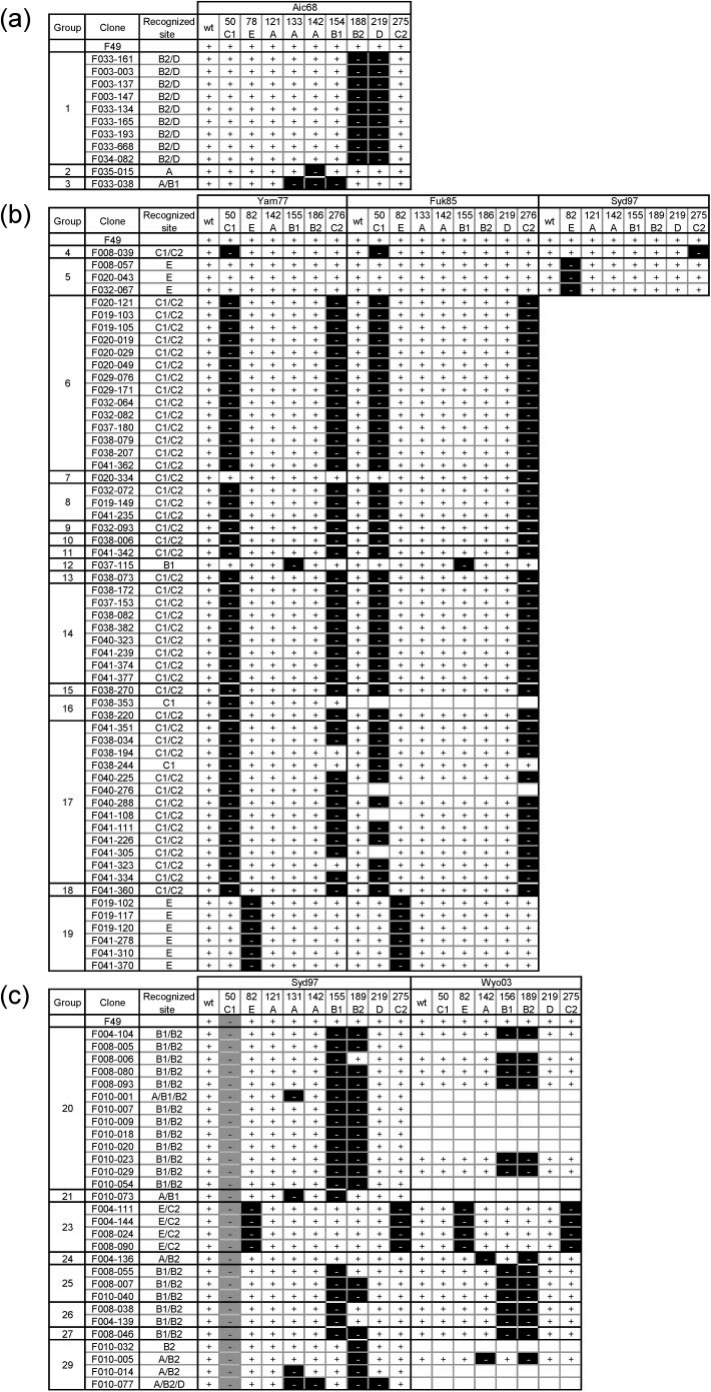
Summary of FCM analyses of 98 mAbs that bound to HA and showed neutralizing activity. Plus (+) indicates that the Ab reacted with HA expressed on the cell surface. Minus (−) indicates that the Ab did not react with the HA expressed on the cell surface at all. Blanks indicate that the analysis was not performed. Recognized site: the location of the epitope recognized by respective clones. (a) The first set of clones, (b) the second set and (c) the third set.

The HA of the Aichi/68 strain and the chimaeric HAs were used to identify the epitopes of the clones in the first set. The HAs of Yamanashi/77 and Fukuoka/85 and their chimaeric HAs were used to identify the epitopes of the clones in the second set. The HAs of Sydney/97 and Wyoming/2003 and their chimaeric HAs were used to identify the epitopes of the clones in the third set. Numerous FCM analyses were systematically performed, and the summary is shown in Fig. 4. The FCM results were judged to be positive, indicating that the Ab bound to the HA expressed on the cell surface, or negative, indicating that the Ab did not bind to the HA.

All nine clones in group 1 did not bind to either A68/188B2 or A68/219D but did bind to the other seven chimaeric HAs. Therefore, we concluded that they bound to site B2/D. Since F035-015 of group 2 did not bind to A68/142A but did bind to the other eight chimaeric HAs, including A68/121A and A68/133A, we concluded that it bound to the C-terminal side of site A. On the other hand, F033-038 in group 3 did not bind to A68/133A, A68/142A or A68/154B1, but it did bind to the other six chimaeric HAs. Therefore, we concluded that it bound to site A/B1 (Fig. 4a).

Since clones in groups 4 and 5 bound to the HAs of 1977–1999 strains, those of Sydney/97 were also used in the FCM analysis in addition to Yamanashi/77 and Fukuoka/85. F008-039 in group 4 did not bind to Y77/50C1, F85/50C1 or S97/275C2 but did bind to all of the other Yamanashi/77, Fukuoka/85 and Sydney/97 chimaeric HAs tested. Although it bound to Y77/276C2 and F85/276C2, site C2 of these chimaeric HAs was changed from GTCSC (1977 and 1985 type) to GKCNS (1997 type), and F008-039 bound not only to Yamanashi/77 and Fukuoka/85 HAs but also to Sydney/97 HA. Thus, we concluded that F008-039 bound to site C1/C2. While the results from the analysis of Sydney/97 indicated that three clones of group 5 bound to site E, they bound to all of the chimaeric HAs of Yamanashi/77 and Fukuoka/85 including site E chimaera. However, the clones of group 5 bound to the HAs of the 1977–1999 strains. Therefore, if they bound to site E, they could bind to the HA with the sequence from the 78th to 83rd residues, GFQNEK (1977 and 1985 type) or GFQNKE (1997 type), but could not bind to either VFQNET (1968 type) or GFQNKK (2003 type) (Fig. 1). The reason why they bound to chimaeric HAs Y77/82E and F85/82E was that site E sequence had been changed in both chimaeras but designed to be the 1997 type. Thus, we concluded that group 5 clones bind to site E (Fig. 4b).

All 14 clones in group 6 bound to site C1/C2. F020-334 in group 7 was judged to bind to site C2 since the interaction between F020-334 and F85/276C2 was negative. On the other hand, the interaction between F020-334 and Y77/276C2 was judged to be positive. However, the interaction between F020-334 and site C1 and C2-chimaeric HA of Yamanashi/77 and Fukuoka/85 strains was largely decreased according to the raw data. Therefore, we concluded that F020-334 bound to site C1/C2. F037-115 of group 12 bound to site B1, and six clones of group 19 recognized site E. The remaining clones, which were six clones in groups 8–11 and 26 clones in groups 13–18, were judged to bind to site C1/C2 (Fig. 4b).

The clones in the third set recognized a rather diverse set of epitopes (Fig. 4c). The majority of clones in groups 20 and 25–27 were judged to bind site to B1/B2. F010-073 of group 21 bound to site A/B1, and clones of group 23 bound to site E/C2. F004-136 of group 24 did not bind to W03/142A or W03/189B2 but did bind to all five other chimaeras. Therefore, we concluded that it recognized site A/B2, although the results from Sydney/97 chimaeras were rather marginal. The epitope recognized by the clones belonging to 29 did not appear to be strictly fixed, but we speculate that they might recognize site B2 and the surrounding region.

### Further validation of the EMAC method

**Fig. 5.**
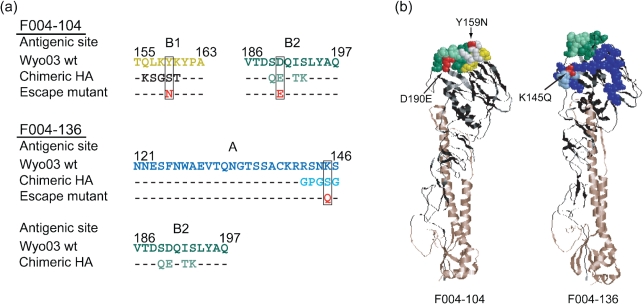
Comparison of the sites assigned by the EMAC method with those determined by the isolation of escape mutants. (a) Amino acid sequences of Wyoming/03 HA, those replaced in the chimaeric HA and those in the escape mutants are indicated. Bars indicate the same amino acid as those of Wyo/03. (b) Locations of the epitopes identified by the EMAC method and by the isolation of escape mutants are indicated in the 3D structure of HA. The illustrations were constructed by using the structure of H3 HA [Protein Data Bank (PDB) accession code 1HA0] according to molecular graphics viewer RasMol 2.7.5. Amino acids indicated in various colours correspond to those used in (a).

Traditionally, an analysis of escape mutants is used to identify epitopes. We compared the results obtained by the EMAC method with the results from an analysis of escape mutants, as indicated in Fig. 5. The epitope recognized by F004-104 of group 20 and F004-136 of group 24 had been revealed by the analysis of escape mutants (Okada *et al.*, 2010). Escape mutants were isolated by incubating A/Wyoming/3/2003 viruses with IgG_1_ Ab. In the case of F004-104, two types of variation, Y159N and D190E, were identified, and the 159th and 190th residues were localized in sites B1 and B2, respectively (Fig. 5a). According to the EMAC method F004-104 did not react with either W03/156B1 or W03/189B2 (Fig. 4c); thus, the epitope recognized by the clone was mapped to site B1/B2. In the escape mutants isolated in the presence of F004-136, the mutation was identified to be K145Q. According to the EMAC method, F004-136 did not react with either W03/142A or W03/189B2 (Fig. 4c); thus, the epitope recognized by the clone was mapped to site A/B2 (Fig. 5a). As indicated in Fig. 5b, the amino acid residues that were mutated in the escape mutants were located in the sites that were changed in the chimaeras.

### Haemagglutination inhibition (HI) activity

Since the HI activity has been routinely measured by many researchers (Smith *et al.*, 2004; Wang *et al.*, 1986), we examined the HI activity of the 98 mAbs whose epitopes were analysed by the EMAC method (data not shown). The results can be summarized as follows: (i) the majority of the clones recognizing A and/or B site had strong HI activity; (ii) the clones recognizing site E generally showed HI activity but not strong activity; and (iii) the clones recognizing site C did not show any HI activity. Since HI activity is the ability of Ab to block the interaction between HA and sialic acid, the regularity observed is reasonable.

## DISCUSSION

**Fig. 6.**
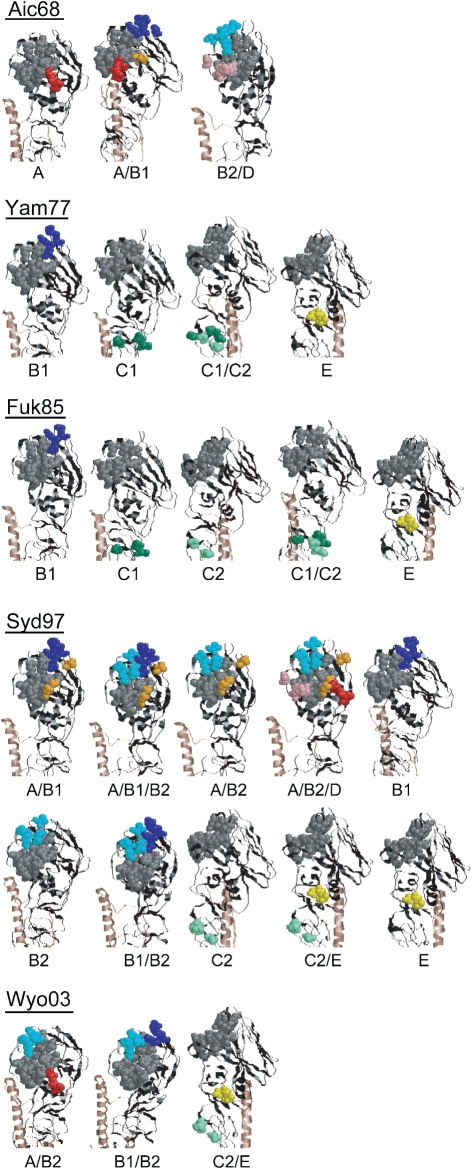
The antigenic site of HA recognized by 98 mAbs that showed binding and neutralizing activity against H3 influenza viruses. Illustrations of the 3D model were constructed in the same way as described in Fig. 5(b). The amino acids that formed a receptor-binding site are marked in grey. When loss of Ab binding was observed in some chimaeric HA, the amino acids replaced in the chimaera were marked in the same way as in Fig. 1(b).

In a previous paper (Okada *et al.*, 2010), we reported the strain specificity of clones that could represent the *in vivo* repertoire of neutralizing mAbs against H3N2 influenza viruses from a donor born in 1960. These clones could be divided into three major groups showing distinct strain specificity: 1968–1973, 1977–1993 and 1997–2003. In the present study, we determined the location of epitopes recognized by these mAbs. We developed a new method, EMAC, that allowed us to comprehensively identify the location of epitopes recognized by numerous clones. While the EMAC method does not provide direct evidence showing the location of an epitope, it is highly plausible that the epitopes identified by this method are indeed correct. All the locations identified were superimposed on the 3D structure of the membrane-distal half of HA, which includes five antigenic sites. Fig. 6 illustrates the sites recognized by mAbs with neutralizing activity that were isolated from the donor in June 2004. As indicated in this figure, all five antigenic sites were immunogenic in the donor.

A set of B cells producing Abs that can react with viruses are generated by immunization through infection and/or vaccination. Afterwards they will take various courses under further stimulation with the Ags. Many B cells disappear while others become memory cells. Since all of the clones that were analysed in the present study were highly mutated (see Table 2 in Okada *et al.*, 2010), it is likely that they represent the repertoire of memory B cells. In our experiments, we analysed the Ab repertoire formed by B cells isolated from the peripheral blood of a donor in June 2004. Therefore, the clones classified into groups 20–29 should reflect the Ab repertoire generated by infection and/or vaccination during the period 1997–2003. The epitopes mapped by using Sydney/97 and Wyoming/2003 HAs were distributed in all five antigenic sites. On the other hand, the majority of the clones that were classified into groups 4–19 recognized the antigenic site C. The presence of two kinds of memory B cells, relatively short-lived cells and long-lived cells, may explain the mechanism for generating such differences. For example, the clones that neutralized the 1968–1973 strains would not have been stimulated by Ags after viruses with which they were unable to react became dominant in the circulation. Despite the lack of stimulation, these memory B cells remained in the donor's body for more than 30 years. Therefore, it appears that some memory B cells persist in the body for a long time; in the most extreme case, memory B cells could persist for a lifetime. The donor had the chance to be infected almost every year by novel influenza viruses that had drifted away from previous viruses. Therefore, some pre-existing memory B cells produced Abs that could neutralize the novel viruses, but others produced Abs that could not neutralize the novel viruses. It might be possible that even if B cells become memory cells, their numbers gradually decrease, and eventually they disappear from the body without further Ag stimulation. In Okada *et al*. (2010), we compared 50 aa of the five antigenic sites of 12 strains whose amino acids were variable during the period 1968–2004. While no mutations were introduced into site C between 1977 and 1993, many mutations were introduced into sites A and B. According to this hypothesis the stability of the epitopes would greatly affect the fate of memory B cells.

Another situation should also be considered to gain insight into memory B cells. When the donor was infected with novel viruses, the generation of new B cells and the activation of pre-existing memory B cells occurred simultaneously. New amino acids were always found at several residues in a newly emergent strain with the exception of the 1970 strain (see Fig. 5 in Okada *et al.*, 2010). When the amino acid sequences were compared between the 1970 and 1973 strains, different amino acids were found at seven residues in the five antigenic sites. However, there were no clones that neutralized the 1973 strain but did not neutralize the 1968 and 1970 strains. It is possible that such clones were generated by infection with the 1973 virus but they did not become long-lived memory B cells. Thus, we speculate that the fate of newly generated B cells is determined by whether Abs produced by pre-existing memory B cells are able to neutralize the newly emergent viruses. As long as the pre-existing memory B cells produce neutralizing Abs effective against novel viruses, it might be difficult for newly generated B cells against the novel viruses to become long-lived memory cells. The majority of the clones were classified into three groups with distinct strain specificity, 1968–1973, 1977–1993 and 1997–2003; we suspect that this is because the largest change in antigenic epitopes was observed between 1973 and 1977 as well as between 1993 and 1997. The Abs produced by pre-existing B cells could not effectively neutralize the viruses that were newly emergent between 1973 and 1977 as well as between 1993 and 1997. Although there could be other possible explanations, an analysis of another Ab library from a donor who was born in 1944 gave essentially the same results as those described in this paper (unpublished results).

According to these hypotheses, the reason why memory B cells producing Abs that recognized site C became dominant during 1977 and 1993 could be explained as follows. When amino acid sequences were compared between the 1973 and 1977 strains, different amino acids were found at 15 residues in the seven antigenic sites (in this case, B1 and B2 as well as C1 and C2 were counted as different sites). Therefore, it was possible that most of the Abs produced by pre-existing B cells were not effective against the newly emergent viruses. Then, many kinds of B cells that produced neutralizing Abs against the emergent viruses were generated by infection and played roles in protection against the viruses. Since various sites should have been immunogenic, the epitopes recognized by such Abs were located not only at site C but also at sites A and B. Initially, all of these B cells had equal chances of becoming long-lived memory B cells. However, while mutations were introduced into various residues in sites A, B1 and B2 during 1977–1993, no difference was generated in sites C1 and C2. Therefore, the memory B cells that produced Abs against sites C1 and C2 were stimulated every year by infection. On the other hand, the growth of B cells producing Abs that bound to site A or B was not enhanced after the introduction of mutations into the relevant residues, and the numbers of these B cells gradually decreased.

In the present analysis, a set of Abs recognizing site C played major roles in protection of a donor against the influenza virus for around 20 years. Since we have analysed B cells from only two donors to date, it is premature to generalize our observations to humans. Recently, however, several papers reported the presence of a neutralizing epitope that was commonly found among the H1 and H5 strains (Ekiert *et al.*, 2009; Kashyap *et al.*, 2008; Sui *et al.*, 2009; Throsby *et al.*, 2008). If the epitope whose sequence has been highly conserved is generally immunogenic in humans, it might be possible and desirable to develop a new type of vaccine that can specifically stimulate the growth of B cells that produce Abs against such a neutralizing epitope. We also found that Abs recognizing site C do not show HI activity. Traditionally, the global influenza surveillance network has adopted the HI assay for characterization of the antigenic properties of influenza viruses. Since we could not isolate escape mutants under the presence of any of the mAbs that recognize site C (unpublished results), it is possible that the alteration of sequences in site C is a much rarer event than that of sites A and B. Thus, we suggest that when the antigenic and genetic evolution of influenza viruses is examined, the contribution of Abs that recognize site C should be considered (Koelle *et al.*, 2006; Smith *et al.*, 2004).

## METHODS

### Viruses.

Twelve influenza A vaccine strains of type H3N2 (A/Aichi/2/68, A/Fukuoka/1/70, A/Tokyo/6/73, A/Yamanashi/2/77, A/Niigata/102/81, A/Fukuoka/C29/85, A/Guizhou/54/89, A/Kita-kyushu/159/93, A/Sydney/5/97, A/Panama/2007/99, A/Wyoming/3/2003 and A/New York/55/2004) were used in this study.

### Preparation of mAbs.

The human mAbs with neutralizing activity used in this study were described previously (Okada *et al.*, 2010). The Fab-PP (P denotes a single Fc-binding domain of protein A) form of Abs (Ito & Kurosawa, 1993) was purified with IgG Sepharose (GE Healthcare) and used in FCM analysis as well as in the HI assay. Alexa 488-anti-human IgG and Alexa 488-anti-mouse IgG used as the second Abs in FCM were purchased from Invitrogen. IgG was prepared by using a high expression vector (Bebbington *et al.*, 1992) and purified with Protein A Sepharose (GE Healthcare). Purified IgGs were used in the HI assay.

### Construction of plasmid DNAs for cell-surface expression of HA and chimaeric HAs.

Details are described in Supplementary Fig. S1 and the Supplementary Methods (available in JGV Online).

### Cell culture and transfection.

293T cells were maintained in Dulbecco's modified Eagle's medium (Wako) containing 10 % FBS and penicillin/streptomycin (Gibco). For FCM analysis, 293T cells in a 150 mm dish were transfected with 30 μg plasmid DNA (HA-chimaera expression vector) and 75 μl Lipofectamine LTX (Invitrogen) and were assayed 24 h after transfection. Cell-surface HA expression was verified by using anti-HA Ab F49 (Ueda *et al.*, 1998).

### Haemadsorption assay.

Haemadsorption activity of HA expressed on the cell surface was checked as described previously Nakajima *et al.* (2005) with some modifications. In brief, 0.3 ml of 2 % guinea-pig red blood cells was mixed with 0.3 ml of HA-expressed 293T cell suspension (5×10^5^) in a microtube, and the mixture was rotated slowly at 4 °C. After 1 h, the microtube was allowed to stand for 10–15 min, so that 293T cells were precipitated at the bottom of the microtube while almost all of the free blood cells remained suspended. The suspended blood cells were removed, and the cell pellet was suspended in 1 ml of PBS in 0.05 % NaN_3_. The microtube was allowed to stand for 10–15 min and the suspended blood cells were removed again. The resulting cell pellet was suspended in 40 μl of PBS in 0.05 % NaN_3_, and the complexes of the HA-expressed 293T cells and the blood cells were observed under an optical microscope.

### FCM analysis.

Cells transfected with HA expression vector (5×10^5^ cells per well) were incubated with 2.5 % goat serum in 2.5 % BSA for 30 min, then incubated with 5 μg Fab-PP ml^−1^ or a 1 : 200 dilution of mouse mAb F49 for 1 h. Cells were washed with 2.5 % BSA, followed by incubation with Alexa 488-conjugated anti-human IgG or Alexa 488-conjugated anti-mouse IgG for 1 h. Then, cells were washed with 2.5 % BSA twice and resuspended in PBS at 5×10^5^ cells ml^−1^ for analysis on a FACScan flow cytometer (Cytomics FC 500; Beckman Coulter). HR1-007, a haemorrhagic factor of Habu venom that recognizes the Fab region of the Ab, was used as a negative control for Fab-PP, and a 1 : 200 dilution of mouse serum was used as a negative control for F49. All reactions were performed on ice.

### HI assay.

The HI test was performed as described previously (Okuno *et al.*, 1990). Briefly, serial dilutions of 100 μg purified Fab-PP ml^−1^ or 100 μg purified IgG ml^−1^ in PBS were prepared. Serial dilutions of Fab-PP were preincubated with 4 HA units of virus per well. Guinea-pig red blood cells were added to a final concentration of 0.5 %, and the plate was incubated at room temperature for 30–60 min.

## Supplementary Material

[Supplementary Material]
