# Supplementary material for: Localization of epitopes recognized by monoclonal antibodies that neutralized the H3N2 influenza viruses in man
Source: J Gen Virol. 2011 Feb;92(Pt 2):326–35. doi: 10.1099/vir.0.026419-0 (PMC3081080; doi:10.1099/vir.0.026419-0)
Supplement: [Supplementary Material] [file supp_92_2_326__index.html]

 Localization of epitopes recognized by monoclonal antibodies that neutralized the H3N2 influenza viruses in man -- Okada et al. 92 (2): 326 Data Supplement - Supplementary Material -- Journal of General Virology

## 

### Localization of epitopes recognized by monoclonal antibodies that neutralized the H3N2 influenza viruses in man, by J. Okada, N. Ohshima, R. Kubota-Koketsu, Y. Iba, S. Ota, W. Takase, T. Yoshikawa, T. Ishikawa, Y. Asano, Y. Okuno and Y. Kurosawa

*Journal of General Virology* vol. **92**, part 2, pp. 326 - 335

**Supplementary Methods.** Construction of plasmid DNAs for cell-surface expression of HA and chimaeric HAs

**Supplementary Fig. S1.** Nucleotide sequences of the primers used in PCR for construction of chimaeric HA genes   
  
 [Single PDF file] (130 KB)
