# Supplementary material for: Localization of epitopes recognized by monoclonal antibodies that neutralized the H3N2 influenza viruses in man
Source: J Gen Virol. 2011 Feb;92(Pt 2):326–35. doi: 10.1099/vir.0.026419-0 (PMC3081080; doi:10.1099/vir.0.026419-0)
Supplement: [Supplementary Material] [file supp_92_2_326__1.pdf]

Supplementary Methods

Construction of plasmid DNAs for cell-surface expression of HA and chimaeric HAs.

The pNOW3 expression vector (Ohtani *et al.*, 1999) was donated by Dr Y. Suzuki (Osaka Prefecture Institute of Public Health). First, the DNA fragment encoding HA of the Aichi/68 strain was inserted into the *Not*I/*Xba*I site of pNOW3. However, the *Xba*I site was replaced by the *Nhe*I site to facilitate construction of vectors that expressed HAs of the other four strains. This plasmid was termed pNOW3/Aic68HA. RNAs were extracted from four kinds of influenza viruses: A/Yamanashi/2/77, A/Fukuoka/C29/85, A/Sydney/5/97 and A/Wyoming/3/2003. DNA fragments encoding entire HA molecules were amplified by RT-PCR by using virus RNA as a template and the following two primers: HA-F primer: 5′-TATGCGGCCGCATGAAGACTATCATTGCTTTGAGCTAC (*Not*I site underlined); and HA-R primer: 5′-CTAGCTAGCTCAAATGCAAATGTTGCACCTAATG (*Nhe*I site underlined). The amplified fragments were inserted into the *Not*I/*Nhe*I site of pNOW3/Aic68HA, resulting in four kinds of HA-expressing plasmid DNAs: pNOW3/Yam77HA, pNOW3/Fuk85HA, pNOW3/Syd97HA and pNOW3/Wyo03HA.

DNAs encoding chimaeric HA variant proteins were prepared according to the following procedure. Using the five kinds of HA-expressing plasmid DNAs as template, artificial genes encoding chimaeric HAs in which one of the seven sites (A, B1, B2, C1, C2, D and E) was replaced by some other strain-derived sequence were generated by two-step PCR. Two kinds of primers covering the site that was planned to be replaced were prepared. One primer was a forward (F) primer, and the other was a reverse (R) primer, and both primers could form a hybrid. In the present study, we constructed nine, eight, eight, nine and seven kinds of chimaeric HAs for the Aichi/68, Yamanashi/77, Fukuoka/85, Sydney/97 and Wyoming/2003 strains, respectively. Therefore, 41 pairs of F and R primers were prepared. The nucleotide sequences of these primers are listed in Supplementary Fig. S1. Other than these primers, two common primers were prepared: pNOW3F: 5′-TTGACGCAAATGGGCGGTAG; and pNOW3R: 5′-TAGGAAAGGACAGTGGGAGTGG. They correspond to the upstream sequence of the *Not*I site in pNOW3 and the downstream sequence of the *Nhe*I site in pNOW3, respectively. For example, to make a chimaeric HA, A68/50C1, that was a variant of Aichi/68HA whose site C1 was replaced, the first PCR was performed as follows. By using pNOW3/Aic68HA as a template and pNOW3F and A68/50C1/R primers, the

Okada, J., Ohshima, N., Kubota-Koketsu, R., Iba, Y., Ota, S., Takase, W., Yoshikawa, T., Ishikawa, T., Asano, Y., Okuno, Y. and Kurosawa, Y. (2011). Localization of epitopes recognized by monoclonal antibodies that neutralized the H3N2 influenza viruses in man. *J Gen Virol* **92**, 326–335.

DNA fragment sandwiched by the two primers was amplified. Separately, by using pNOW3/Aic68HA as a template and A68/50C1/F and pNOW3R primers, the DNA fragment sandwiched by the two primers was amplified. Both amplified fragments were purified by gel electrophoresis. Then, the second PCR was performed. After they were mixed, DNA encoding the entire HA region was amplified by using pNOW3F and pNOW3R primers. The DNA encoding the entire HA containing a replaced portion was inserted into the *Not*I/*Nhe*I portion of the pNOW3 vector. After construction of the plasmid, the DNA nucleotide sequence of the HA-coding region was determined. We prepared 41 kinds of plasmid DNAs essentially in the same way as described here. The DNA sequences indicated that there was no case in which a wrong nucleotide was incorporated during the construction of plasmid DNAs.

Supplementary Reference

Ohtani, K., Suzuki, Y., Eda, S., Kawai, T., Kase, T., Keshi, H., Sakai, Y., Yamamoto, S., Sakamoto, T. & Wakamiya, N. (1999). High-level and effective production of human mannan-binding lectin (MBL) in Chinese hamster ovary (CHO) cells. *J Immunol Methods* **222**, 135–144. Medline

---

Supplementary Fig. S1. Nucleotide sequences of the primers used in PCR for construction of chimaeric HA genes. Codons encoding amino acids newly replaced in the chimaeric HA are indicated in red in the forward (F) primers. Anticodons encoding amino acids newly generated in the chimaeric HA are indicated in blue in the reverse (R) primers.

Yam77

Forward primer		Reverse primer	
A68/50C1/F	5'-ggGGAatatgcGACAGTcctcatCAAatccttgatggaGAAAACtgcacactgatagatg	A68/50C1/R	5'-caGTTTTCtccatcaaggatTTGatgaggACTGTCgcatatTCCccccgttgaggag
A68/78E/F	5'-gatGGTtttcaaaatAAGGAAtgggacctt	A68/78E/R	5'-ccaTTCCTTattttgaaaACCatcacaatg
A68/121A/F	5'-ttAACAATgagAGTttcAATtggGCTggggtcactcag	A68/121A/R	5'-ccAGCccaATTgaaACTctcATTGTTaaactccagagtgcct
A68/133A/F	5'-ggtcactcagGATgggggaagcaatg	A68/133A/R	5'-cccATCctgagtgaccccagtc
A68/142A/F	5'-gcaaaaggAGATCTAATAAGAGTtttttc	A68/142A/R	5'-aACTCTTATTAGATCTccttttgcaagcat
A68/154B1/F	5'-ggCTGaccCACTTAAAATACAAAtatccaGCGctgaacgtgactatg	A68/154B1/R	5'-agCGCtggataTTTGTATTTTAAGTGggtCAGccagttcagtctactg
A68/188B2/F	5'-cgGACTCAGATcaaATCagcctgtatGCTcaagcatcagggaga	A68/188B2/R	5'-tgAGCatacaggctGATttgATCTGAGTCcgtgctcgggtgg
A68/219D/F	5'-TACagacccCGGgtaaggGATATCtctagt	A68/219D/R	5'-aGATATCccttacCCGgggtctGTAcccga
A68/275C2/F	5'-ttGGTAAGtgtAATtctgaatgcatcactcc	A68/275C2/R	5'-gaATTacaCTTACCaataggtgcatctgac

Fuk85

Forward primer		Reverse primer	
Y77/50C1/F	5'-gtAAAatatgcAACAATcctcatcgaatccttgatg	Y77/50C1/R	5'-gaggATTGTTgcatatTTTacctgttgaggaactc
Y77/82E/F	5'-caaaatAAGGAAtgggacctttttgttgaac	Y77/82E/R	5'-ggtcccaTTCCTTattttgaaagccatcacaatg
Y77/142A/F	5'-ggAGATCTAATAAGagtttcttcagtagactgaa	Y77/142A/R	5'-gaaactCTTATTAGATCTccttttgcaagcacag
Y77/155B1/F	5'-ggttgACCCAATTAAAATACaaatatccagtgctgaac	Y77/155B1/R	5'-GTATTTTAATTGGGTcaaccagttcagtctac
Y77/186B2/F	5'-GTCacggacAGTGACcaaaccaacctatatgttcaag	Y77/186B2/R	5'-GTCACTgtccgtGACcgggtggtgaacc
Y77/276C2/F	5'-ggcAAGtgcAATtctgaatgcatcactcc	Y77/276C2/R	5'-cagaATTgcaCTTgccaataggtgcatc

Syd97

Forward primer		Reverse primer	
F85/50C1/F	5'-caggtAAAatatgcAACAATcctcaccgaatccttg	F85/50C1/R	5'-gaggATTGTTgcatatTTTacctgttgaggaactctg
F85/82E/F	5'-ccaaaatAAGGAAtgggacctttttgttgaacg	F85/82E/R	5'-ggtcccaTTCCTTattttggaagccatcacaatg
F85/133A/F	5'-agAATgggACAagcTCTgcttgcaaaaggggatc	F85/133A/R	5'-gcAGAgctTGTcccATTctgagtgactccagtc
F85/142A/F	5'-ggCGAtctAATAAGagtttcttcagtagattgaattg	F85/142A/R	5'-gaaactCTTATTagaTCGccttttgcaagcatagc
F85/155B1/F	5'-gaattggttgACCCAATTAAAAtacaaatatccagcgctg	F85/155B1/R	5'-ggatatttgtaTTTTAATTGGGTcaaccaattcaatctactgaa
F85/186B2/F	5'-cgGTCacggacAGCGACcaaaccaaactatatgttcg	F85/186B2/R	5'-tgGTCGCTgtccgtGACcgggtggtgaacc
F85/219D/F	5'-ggTATagacccCGGgtaaggggtctg	F85/219D/R	5'-acCCGgggtctATAcccgatattcgggatt
F85/276C2/F	5'-ggcAAGtgcAATtctgaatgcattactccaaa	F85/276C2/R	5'-ttcagaATTgcaCTTgccaataggtgcatc

Wyo03

Forward primer		Reverse primer	
S97/50C1/F	5'-caggtAAAatatgcAACAACcctcaccgaatc	S97/50C1/R	5'-gGTTGTTgcatatTTTacctgttgaggaactc
S97/82E/F	5'-caaaatGAGACAtgggacctttttgttgaac	S97/82E/R	5'-ggtcccaTGTCTCattttggaagccatcacaat
S97/121A/F	5'-ATCACTgaaGGCttcACTtggactggagtc	S97/121A/R	5'-AGTgaaGCCttcAGTGATaaactccagggtgc
S97/131A/F	5'-cACTcagaatggaGGAagcAATgcttgcaaaaggagttc	S97/131A/R	5'-cATTgctTCCtccattctgAGTgactccagtc
S97/142A/F	5'-gGGTCCTGGTAGTGGTttctttagtagattgaattgg	S97/142A/R	5'-aACCACTACCAGGACCccttttgcaagcatagc
S97/155B1/F	5'-gACCAAATCAGGATCCACAtatccagcactgaacg	S97/155B1/R	5'-TGTGGATCCTGATTTGGTcaaccaattcaatctactaaag
S97/189B2/F	5'-cCAGGAAcaaaccagcTTAtatGTTcaagcatcagggag	S97/189B2/R	5'-AACataTAAgctggtttgTTCCTGgtccgtactcgg
S97/219D/F	5'-aTATagacccCGGgtaaggGATatctccagcagaataag	S97/219D/R	5'-ATCccttacCCGgggtctATAtccgatattcgggattac
S97/275C2/F	5'-cattGACACAtgcATTtctgaatgcatcactcc	S97/275C2/R	5'-cagaAATgcaTGTGTCaatgggtgcatctga

Forward primer		Reverse primer	
W03/50C1/F	5'-caggtAAAatatgcAACAATcctcatcagatccttgatg	W03/50C1/R	5'-gaggATTGTTgcatatTTTacctgttgaggaactctgaac
W03/82E/F	5'-ccaaaatGAGACAtgggacctttttgttg	W03/82E/R	5'-aggtcccaTGTCTCattttggaagcc
W03/142A/F	5'-gGGACCTGGTAGCGGTttctttagtagattgaattggttg	W03/142A/R	5'-gaaACCGCTACCAGGTCCccttttgcaagcagagcttg
W03/156B1/F	5'-gaccAAATCAGGATCCACAtacccagcattgaacgtgac	W03/156B1/R	5'-ggtaTGTGGATCCTGATTTggtcaaccaattcaatctac
W03/189B2/F	5'-cCAGGAAcaaACCAAGctatatgctcaagcatcagg	W03/189B2/R	5'-catatagCTTGGTttgTTCCTGgtccgtaaccgggtg
W03/219D/F	5'-TCTagacccTGGgtaaggGGTCTCtccagcagaataagcatc	W03/219D/R	5'-GAGACCccttacCCAgggtctAGAtccgatattcgggattacag
W03/275C2/F	5'-ccattGACACAtgcATTtctgaatgcatcactcc	W03/275C2/R	5'-cagaAATgcaTGTGTCaatgggtgcatctgatctc
